# Several types of groupoids induced by two-variable functions

**DOI:** 10.1186/s40064-016-3411-y

**Published:** 2016-10-04

**Authors:** P. J. Allen, Hee Sik Kim, J. Neggers

**Affiliations:** 1 Department of Mathematics, University of Alabama, 35487-0350 Tuscaloosa, AL USA; 2 Research Institute for Natural Sciences, Department of Mathematics, Hanyang University, 04763 Seoul, Korea

**Keywords:** Groupoid, (Twisted) semigroup, Linear groupoid over a field, $n\mathrm{th}$ power property, Homomorphism, Primary 20N02, 20M99

## Abstract

In this paper, we introduce the concept of several types of groupoids related to semigroups, viz., twisted semigroups for which twisted versions of the associative law hold. Thus, if $(X,*)$ is a groupoid and if $\varphi : X^2\rightarrow X^2$ is a function $\varphi (a, b) = (u, v)$, then $(X,*)$ is a left-twisted semigroup with respect to $\varphi$ if for all $a, b, c\in X$, $a*(b*c) = (u*v)*c$. Other types are right-twisted, middle-twisted and their duals, a dual left-twisted semigroup obeying the rule $(a*b)*c = u*(v*c)$ for all $a, b, c\in X$. Besides a number of examples and a discussion of homomorphisms, a class of groupoids of interest is the class of groupoids defined over a field $(X,+, \cdot )$ via a formula $x*y=\lambda x + \mu y$, with $\lambda , \mu \in X$, fixed structure constants. Properties of these groupoids as twisted semigroups are discussed with several results of interest obtained, e.g., that in this setting simultaneous left-twistedness and right-twistedness of $(X,*)$ implies the fact that $(X,*)$ is a semigroup.

## Introduction and preliminaries

Suppose that $X=\mathbf{R}$ is the set of all real numbers and that we consider the binary operation $(\mathbf{R}, -)$ where “−” is the usual subtraction. Then $(x-y)-z\not = x-(y-z) = x-y + z$ in general, i.e., $(\mathbf{R}, -)$ is not a semigroup. Since $(x-y)-z = x-(y-(-z))$, if we define $u:=x, v:=-z$, then we have $(x-y)-z = u-(y-v)$, which looks like that “−” satisfies a version of the associative law in $\mathbf{R}$, i.e., there exists a map $\varphi : \mathbf{R}^2 \rightarrow \mathbf{R}^2$ such that $\varphi (x, z)=(x, -z)=(u, v)$ and $(x-y)-z = u-(y-v)$. Thus, we obtain a “twisted” associated law for $(\mathbf{R}, -)$, with the function $\varphi$ defining the “nature” of the “twisted semigroup” of a particular type. Obviously, a twisted semigroup need not be a semigroup. However, semigroups are twisted semigroups where the twist is $\varphi (x, y) =(x, y)$. Twisted semigroups of several types will be the topic of investigation in the following. As algebraic objects they include many familiar examples of groupoids which are definitely not semigroups but whose study benefits from the approach taken in what follows.

In particular, if $(X,+, \cdot )$ is a field and if $x*y=\lambda x + \mu y, \lambda , \mu \in X$, defines a (linear) product, the resulting class of groupoids has a membership whose structure depends both on the nature of the formula as well as on properties of the field $(X,+,\cdot )$ itself. Several conclusions are obtained. A main result is the conclusion that if $(X,*)$ is a left-twisted semigroup with respect to a map $\varphi _1$ and if $(X,*)$ is a right-twisted semigroup with respect to a map $\varphi _2$, then $(X,*)$ is already a semigroup. Homomorphisms of twisted semigroups of the various types are also discussed, and from a counterexample it follows that the class of dual left-twisted semigroups is not a variety, even though direct products and (groupoid) epimorphic images of any of the types of twisted semigroups are also of at least the same type. Although examples of certain twisted semigroups have long been studied in various settings, the notion of a groupoid $(X,*)$ equipped with a twisting mapping $\varphi : X^2\rightarrow X^2$ to produce a twisted semigroup $(X,*)$ of a certain type, appears to be new. For general references on semigroups we refer to Clifford et al. (1961), Howie (1995).

Kim and Neggers (2008) introduced the notion of *Bin*(*X*), the collection of all groupoids defined on a non-empty set *X*. Given arbitrary groupoids $(X, *)$ and $(X, \bullet )$, we define a product $(X, \Box ):= (X, *)\Box (X, \bullet )$ where $x\Box y := (x*y)\Box (y*x)$ for all $x, y\in X$. They showed that $(Bin(X), \Box )$ is a semigroup and the left zero semigroup on *X* acts as an identity in $(Bin(X), \Box )$. Let $(R, +, \cdot )$ be a commutative ring with identity and let *L*(*R*) denote the collection of all groupoids $(R, *)$ such that, for all $x, y\in R$, $x*y:= ax + by + c$, where $a, b, c\, (\in R)$ are fixed constants. Such a groupoid $(R, *)$ is said to be a *linear groupoid*. They showed that $(L(R), \Box )$ is a semigroup with identity.

Some researchers studied on linear groupoids and quadratic groupoids in several algebras. Neggers et al. (2001) introduced the notion of a *Q*-algebra, and showed that every quadratic *Q*-algebra $(X,*, e), e\in X$, has of the form $x*y= x-y +e$ when *X* is a field with $|X|\ge 3$. Moreover, Kim and So (2012) investigated some properties of $\beta$-algebras and they obtained linear $\beta$-algebras.

## Twisted semigroups

Let $(X,*)$ be a groupoid for which there exists a function $\varphi : X^2\rightarrow X^2$ such that, for all $a, b, c\in X$,

1 $$\begin{aligned} a*(b*c) = (u*v)*c \end{aligned}$$

where $\varphi (a,b) =(u, v)$, i.e., $u=u(a, b), v=v(a, b)$ are functions of two variables. Then $(X,*)$ is said to be a *left-twisted semigroup* with respect to the map $\varphi$. Such a map $\varphi$ is called an *associator function* of the groupoid $(X,*)$.

We may think of a dual equation of (1) as follows:

2 $$\begin{aligned} (a*b)*c = u*(v*c) \end{aligned}$$

where $\varphi (a,b) =(u, v)$, i.e., $u=u(a, b), v=v(a, b)$ are functions of two variables. Then $(X,*)$ is said to be a *dual left-twisted semigroup* with respect to the map $\varphi$. The function $\varphi$ is not necessarily unique.

Suppose we replace the Eq. (1) by

3 $$\begin{aligned} (a*b)*c = a*(u*v) \end{aligned}$$

where $\varphi (b,c) =(u, v)$, i.e., $u=u(b, c), v=v(b, c)$ are functions of two variables. Then $(X,*)$ is said to be a *right-twisted semigroup* with respect to the map $\varphi$.

We may think of a dual equation of (3) as follows:

4 $$\begin{aligned} a*(b*c)= (a*u)*v \end{aligned}$$

where $\varphi (b,c) =(u, v)$, i.e., $u=u(b, c), v=v(b, c)$ are functions of two variables. Then $(X,*)$ is said to be a *dual right-twisted semigroup* with respect to the map $\varphi$.

If we replace the Eq. (1) by

5 $$\begin{aligned} (a*b)*c = u*(b*v) \end{aligned}$$

where $\varphi (a,c) =(u, v)$, i.e., $u=u(a, c), v=v(a, c)$ are functions of two variables. Then $(X,*)$ is said to be a *middle-twisted semigroup* with respect to the map $\varphi$.

We may think of a dual equation of (5) as follows:

6 $$\begin{aligned} a*(b*c)= (u*b)*v \end{aligned}$$

where $\varphi (a,c) =(u, v)$, i.e., $u=u(a, c), v=v(a, c)$ are functions of two variables. Then $(X,*)$ is said to be a *dual middle-twisted semigroup* with respect to the map $\varphi$.

If $\varphi : X^2\rightarrow X^2$ is the identity map $\varphi (a, b)= (a, b)$, then the Eqs. (1–6) reduce to the associative law and thus:

### **Proposition 1**

*If* $(X,*)$ *is a semigroup, then it is a (dual) left(right, middle)-twisted semigroup.*

### *Example 1*

Consider $(\mathbf{R}, -)$, the real numbers $\mathbf R$ with the subtraction operation “−”. Since $a-(b-c)\not = (a-b)-c$, the groupoid $(\mathbf{R}, -)$ is not a semigroup. Consider the expression $((a+1)-b)-(1-c) = (a-b) +c= a-(b-c)$. Thus $\varphi (a,c)= (a+1, 1-c)$ produces $(\mathbf{R}, -)$ as a middle-twisted semigroup which is not a semigroup.

### *Example 2*

Consider $X:=2^A$ where $A\not = \emptyset$. If we define $a*b:= a-b$ for any $a, b\in X$, then $(a*b)*c \not = a*(b*c)$. On the other hand, if we let $\varphi (b,c):=(b\cup c, \emptyset )$, then $(a*b)*c = (a-b)-c = a-(b\cup c)$, and $a*(u*v) = a-(b\cup c - \emptyset )=a-(b\cup c)$, proving that $(X,*)$ is a right-twisted semigroup w.r.t. $\varphi$.

Note that Example 2 is a typical example of a *BCK*-algebra which is also a right-twisted semigroup.

### *Example 3*

In Example 1 if we define $\psi (b, c):=(b+c, 0)$, then $a-((b+c)-0) = (a-b)-c$ and hence it is a right-twisted semigroup with respect to $\psi$.

### *Example 4*

Let $(\mathbf{R}, +, \cdot )$ be a real field. Define a binary operation “$*$” on $\mathbf R$ by

$$\begin{aligned} x*y:= x(x-y), \quad \forall x, y\in \mathbf{R}. \end{aligned}$$

Given elements $a, b, c\in \mathbf{R}$, define a map $\varphi : \mathbf{R}^2 \rightarrow \mathbf{R}^2$ by $\varphi (a, b) := (u, v)$ where

$$\begin{aligned} (u, v):=\left\{ \begin{array}{ll} (0, v) & \quad \hbox {if}\,\, a=b,\\ (0, v) & \quad \hbox {if}\,\, a=0, b\not = 0,\\ (\frac{v^2}{1-v^2}, v) & \quad \hbox {if}\,\, a\not = 0, a\not = b \end{array}\right. \end{aligned}$$

with $v^3 = a(a-b)(v^2 - 1)$. Then it is easy to show that $(a*b)*c = u*(v*c)$. This shows that $(\mathbf{R}, *)$ is a dual left-twisted semigroup with respect to $\varphi$. Moreover, it can be shown that the function $\varphi$ is the unique function for making $(\mathbf{R}, *)$ a dual left-twisted semigroup.

### **Proposition 2**

*There is no map* $\varphi : \mathbf{R}^2\rightarrow \mathbf{R}^2$ *such that* $(\mathbf{R}, -)$ *is a left-twisted semigroup.*

### *Proof*

If we assume that there is a map $\varphi : \mathbf{R}^2\rightarrow \mathbf{R}^2$ such that $(\mathbf{R}, -)$ is a left-twisted semigroup,

7 $$\begin{aligned} (a-b)+c = (u-v) -c \end{aligned}$$

for the map $\varphi (a,b)=(u,v)$. If we let $c:=0$, then $a-b= u-v$. Hence, by (7) we obtain $c=-c$, for all $c\in \mathbf{R}$, a contradiction. $\square$

Note that Proposition 2 shows that $(\mathbf{R}, -)$ is a groupoid which can not be a left-twisted semigroup. Moreover, Proposition 2 shows that not every right(middle)-twisted semigroup is a left-twisted semigroup. Examples 1 and 3 together show that a groupoid can fail to be a semigroup and yet be a middle-twisted as well as a right-twisted semigroup.

### **Theorem 1**

*If a left-twisted semigroup has a right identity element, then it is a semigroup.*

### *Proof*

Let $c:=e$ be the right identity element. Then by (1) we have $a*b = a*(b*e) = (u*v)*e = u*v$ and thus $(u*v)*c = (a*b)*c$, so that $a*(b*c) = (a*b)*c$, i.e., the groupoid is a semigroup as claimed. $\square$

Dually, if a right-twisted semigroup has a left identity element, then it is a semigroup.

Notice that $(\mathbf{R}, -)$ has a right identity element 0, but it is not a semigroup. By Theorem 1 it cannot be left-twisted, as shown in Proposition 2.

### **Proposition 3**

*The groupoid* $(\mathbf{R}, -)$ *is a middle-twisted semigroup with respect to* $\varphi$ *if and only if* $u(a, c) + v(a, c) = a -c$ *for any* $a, c\in \mathbf{R}$.

### *Proof*

Given $a,b, c\in \mathbf{R}$, $(a-b)-c= u-(b-v)$, where $u=u(a, b), v=v(a, b)$, means that $a-b-c = u-b +v$, and hence $u(a, c) + v(a, c) = a -c$. The converse is straightforward. $\square$

If we let $u:=a-\alpha , v:=-c +\alpha$ where $\alpha =\alpha (a, c)$, then $(\mathbf{R}, -)$ is a middle-twisted semigroup.

## Twisted semigroups in a field

Let $X=(X,+,\cdot )$ be a field and $\lambda , \mu \in X$ (not all zero). If we define a binary operation “$*$” on *X* as follows:

$$\begin{aligned} x*y:= \lambda x + \mu y \end{aligned}$$

for any $x, y\in X$, we call such a groupoid $(X,*)$ a *linear groupoid* over a field *X*. We define its associator function $\varphi (a,b) :=(u, v)$, i.e., $u=u(a, b), v=v(a, b)$ are functions of two variables $a, b\in X$.

### *Example 5*

Let $\mathbf{R}=(\mathbf{R},+,\cdot )$ be a real field and $\lambda \not = 0, \mu \in \mathbf{R}$. We define a binary operation “$*$” on $\mathbf{R}$ as follows: $x*y:= \lambda x + \mu y$ for any $x, y\in \mathbf{R}$. If we define a map $\varphi (a,b) :=(\frac{a}{\lambda }, b)$ and $\mu ^2 =\mu$, then $(\mathbf{R},*)$ is a left-twisted semigroup with respect to $\varphi$.

### **Proposition 4**

*Let* $(X,*)$ *be a linear groupoid over a field* *X* *and its associator function defined by* $\varphi (a, b):=(\lambda a, b)$. *If* $(X,*)$ *is a dual left-twisted semigroup with respect to* $\varphi$, *then it has the form* $x*y=\lambda x$ *or* $x*y=\lambda x + y$.

### *Proof*

Since $(X,*)$ is a dual left-twisted semigroup with respect to $\varphi$, $(a*b)*c = \lambda ^2 a + \lambda \mu b + \mu c$ and $u*(v*c) = \lambda ^2 a + \mu \lambda b + \mu ^2 c$ for any $a, b, c\in X$. It follows that $\mu =\mu ^2$, proving the proposition. $\square$

In Proposition 4, if we let $\lambda := 2$ and $\mu :=1$, and we define $\varphi (a,b):=(2a, b)$, then $(X,*)$ is certainly a dual left-twisted semigroup with respect to $\varphi$, but it is not a left-twisted semigroup with respect to $\varphi$, since $a*(b*c)= 2a + 2b + c$ and $(u*v)*c = 8a + 2b + c$.

### *Example 6*

Let $(\mathbf{R}, +, \cdot )$ be a real field. Define a binary operation “$*$” on $\mathbf{R}$ as follows: $x*y:= x + 2y, \forall x, y\in \mathbf{R}$. If we define a map $\varphi (b, c):=(b, \frac{c}{2}), \forall a, b\in \mathbf{R}$, then $(\mathbf{R}, *)$ is a right-twisted semigroup with respect to $\varphi$, but not a (dual) left-twisted semigroup with respect to $\psi (a, b):=(a, \frac{b}{2})$, since $(a*b)*c = a + 2b + 2c$, while $u*(v*c) = a + b + 4c$ and $(u*v)*c = a + b + 2c$.

Note that Examples 5 and 6 show that twisted-semigroups may have role to play in the theory of linear groupoids in various algebraic structures (see Kim and Neggers 2008; Kim and So 2012; Neggers et al. 2001).

### **Proposition 5**

*Let* $$(X,*)$$ *be a linear groupoid over a field* $$(X, +, \cdot )$$, *i.e.,* $$x*y:= \lambda x + \mu y$$ *for all* $$x, y\in X$$ *where* $$\lambda , \mu $$ *are not all zero in* *X*. *If* $$(X,*)$$ *is a right-twisted semigroup with respect to* $$\varphi (b, c) = (u, v)$$, *then it has one of the forms:* (i) $$x*y= x $$; (ii) $$x*y=\mu y$$, $$\mu \not = 0$$ *and* $$u=b, v =\frac{c}{\mu }$$; (iii) $$x*y= x + \mu y$$, $$\mu \not = 0$$ *and* $$u=b, v= \frac{c}{\mu }$$.

### *Proof*

Since $(X,*)$ is a linear groupoid over *X*, there exist $\lambda , \mu \in X$ (not all zero) such that $x*y = \lambda x + \mu y$ for all $x, y\in X$. Let $(X,*)$ be a right-twisted semigroup with respect to $\varphi (b, c) = (u, v)$. Then $(a*b)*c = (\lambda a + \mu b)*c = \lambda (\lambda a + \mu b) + \mu c = \lambda ^2 a + \lambda \mu b + \mu c$ and $a*(u*v) = a*(\lambda u + \mu v) = \lambda a + \mu (\lambda u + \mu v) = \lambda a + \lambda \mu u + \mu ^2 v$. It follows that

8 $$\begin{aligned} \lambda ^2 a + \lambda \mu b +\mu c= \lambda a + \lambda \mu u + \mu ^2 v \end{aligned}$$

Assume that $\mu \not = 0$. If we let $u:= b, v:= \frac{c}{\mu }$, then

9 $$\begin{aligned} \lambda ^2 a + \mu c = \lambda a + \mu c \end{aligned}$$

and hence $\lambda ^2 a =\lambda a$, i.e., $\lambda = 0$ or $\lambda = 1$. If $\lambda = 0$, then $x*y = \mu y$. If $\lambda \not = 0$, then $x*y = x + \mu y$. Assume $\mu = 0$. Then $x*y = \lambda x$ and hence

10 $$\begin{aligned} \lambda a = a*(u*v) = (a*b) * c = \lambda ^2 a \end{aligned}$$

It follows that $\lambda = 0$ or $\lambda = 1$, which shows that $x*y = x$, since $(X,*)$ is a linear groupoid. $\square$

### **Proposition 6**

*Let* $(X,*)$ *be a linear groupoid over a field X. If we define a map* $\varphi (b, c):=(b, \mu c), \forall b, c\in X$, *then* $(X,*)$ *is a dual right-twisted semigroup with respect to* $\varphi$ *when* $\lambda ^2 = \lambda$.

### *Proof*

Straightforward. $$\square $$

### **Theorem 2**

*Let* $(X,*)$ *be a linear groupoid over a field* *X*, *i.e.,* $x*y:=\lambda x + \mu y$ *for all* $x, y\in X$ *and let* $\lambda \mu \not = 0$. *If* $(X,*)$ *is both a left-twisted semigroup with respect to a map* $\varphi (a, b) = (\frac{a}{\lambda }, b)$ *and a right-twisted semigroup with respect to a map* $\psi (b, c) = (b, \frac{c}{\mu })$, *then* $(X,*)$ *is an additive group of the field* *X*.

### *Proof*

Since it is a left-twisted semigroup, given $a, b, c\in X$, $a*(b*c) = \lambda a + \lambda \mu b + \mu ^2 c$ and $(u*v)*c = \lambda ^2 u + \lambda \mu v + \mu c$ for some $u= u(a, b), v=v(a, b)$. Hence we have

11 $$\begin{aligned} \lambda a + \lambda \mu b + \mu ^2 c = \lambda ^2 u + \lambda \mu v + \mu c \end{aligned}$$

Similarly, since it is a right-twisted semigroup, we have

12 $$\begin{aligned} \lambda ^2 a + \lambda \mu b + \mu c = \lambda a + \lambda \mu u^{\prime } + \mu ^2 v^{\prime } \end{aligned}$$

for some $u^{\prime } = u^{\prime }(a, b), v^{\prime } = v^{\prime }(a, b)$. If we put $u:= \frac{a}{\lambda }, v:= b$ in (11), then $\mu ^2 c = \mu c$. It follows that $\mu =1$. If we put $u^{\prime }:= b, v^{\prime }:= \frac{c}{\mu }$ in (12), then $\lambda ^2 a =\lambda a$, proving that $\lambda = 1$. Hence $x*y = x + y$, i.e., $(X,*)$ is an additive group of the field *X*. $\square$

### **Proposition 7**

*Let* $(X,*)$ *be a linear groupoid over a field* *X*, *i.e.,* $x*y =\lambda x + \mu y$ *for all* $x, y\in X$ *where* $\lambda , \mu$ *are not all zero. If we define a map* $\varphi (a, c) :=(\lambda a, \frac{1}{\mu }c), \forall a,c \in X$ *where* $\mu \not = 0$, *then* $(X, *)$ *is a middle-twisted semigroup with respect to* $\varphi$.

### *Proof*

Straightforward. $$\square $$

### *Example 7*

Let $(\mathbf{R}, +, \cdot )$ be a real field. Define a binary operation “$*$” on $\mathbf{R}$ by $x*y := x-y, \forall x, y\in \mathbf{R}$ and define a map $\varphi (a, c) :=(a, -c), \forall a, c \in \mathbf{R}$. Then $(\mathbf{R}, *)$ is a dual middle-twisted semigroup with respect to $\varphi$.

## Twisted semigroups on groups

### **Proposition 8**

*Let* $(G, \cdot )$ *be a group and* $n\in \mathbf{N}$ *(fixed). Define a binary operation* “$*$” on *G* by $a*b:= a^nb, \forall a, b\in G$. *If we define a map* $\varphi (a, b):= (a^nb, e), \forall a, b\in G$ *where* *e* *is the identity of* *G*, *then* $(G,*)$ *is a dual left-twisted semigroup with respect to* $\varphi$.

### *Proof*

Given $a, b, c\in G$, we have $(a*b)*c = (a^nb)^nc$. Since $\varphi (a, b):= (a^nb, e)$, if we let $u:= a^nb, v:=e$, then $u*(v*c)= u^n(v^nc)= (a^nb)^n(e^nc) = (a^nb)^nc$, proving the proposition. $\square$

Let *n* be a natural number. A group $(G, \cdot )$ is said to have the $n{\rm th}$ *power property* if there are $a, b\in G$ such that $a^nb^n = x^n$ has no solution *x* in *G*. For example, consider the dihedral group $D_4=\{r_0, r_1, r_2, r_3, h, v, d, t\}$ in (Hungerford 1990, p.158). It is easy to show that $r_3^3\cdot v^3 = d$, but there is no element $x\in D_4$ such that $x^3= d$.

### **Proposition 9**

*Let* $(G, \cdot )$ *be a group having the* $n^{th}$ *power property. Define a binary operation* “$*$” *on* *G* *by* $a*b:= a^nb, \forall a, b\in G$. *Then* $(G,*)$ *is not a left-twisted semigroup with respect to any mapping* $\varphi$.

### *Proof*

Assume that $(G,*)$ is a left-twisted semigroup with respect to some mapping $\varphi$. Then for any $a, b, c\in G$, there exist $u= u(a, b), v = v(a, b)$ in *G* such that $\varphi (a, b) = (u, v)$ and $a*(b*c) = (u*v)*c$. This means that $a^nb^nc = (u^nc)^nc$. Since $(G, \cdot )$ is a group, we obtain $a^nb^n = (u^nv)^n$. If we let $x:= u^nv$, then $a^nb^n = x^n$ has a solution $x=u^nv$, a contradiction. $\square$

### **Proposition 10**

*Let* $(G, \cdot )$ *be a group having the* $n^{th}$ *power property. Define a binary operation* “$*$” *on* *G* *by* $a*b:= ab^n, \forall a, b\in G$. *Then* $(G,*)$ *can not be a right-twisted semigroup with respect to any mapping* $\varphi$.

### *Proof*

Assume that $(G,*)$ is a right-twisted semigroup with respect to $\varphi$. Since $(G, \cdot )$ has the $n^{th}$ power property, there are $b, c\in G$ such that $b^nc^n = x^n$ has no solution in *G*. Since $(G, *)$ is a right-twisted semigroup, for any $a\in G$, there exist $u=u(b, c), v=v(b, c)$ in *G* such that $(a*b)*c = a*(u*v)$. Hence $(ab^n)c^n = a(uv^n)^n$, i.e., $b^nc^n = (uv^n)^n$. If we let $x:= uv^n$, then $b^nc^n = x^n$ has a solution, a contradiction. $\square$

## Homomorphisms of twisted semigroups

### **Theorem 3**

*Let* $(X, *)$ *be a left-twisted semigroup with respect to a map* $\varphi : X^2\rightarrow X^2$. *If* $f: (X,*)\rightarrow (Y, \bullet )$ *is an* *epimorphism of groupoids, i.e.,* $f(x*y)= f(x)\bullet f(y), \forall x, y\in X$, *then there exists a map* $\psi : Y^2\rightarrow Y^2$ *such that* $(Y, \bullet )$ *is a left-twisted semigroup with respect to the map* $\psi$.

### *Proof*

Given $\alpha , \beta \in Y$, since $f: X\rightarrow Y$ is onto, there exist $a, b\in X$ such that $\alpha = f(a), \beta = f(b)$. Since $(X, *)$ is a left-twisted semigroup with respect to a map $\varphi$, for all $c\in X$, there exist $u_0= u_0(a, b), v_0 = v_0(a, b)\in X$ such that $a*(b*c) = (u_0 * v_0)*c$ and $\varphi (a, b) = (u_0, v_0)$. We define a set $\Gamma _{(\alpha , \beta )}$ as follows:

$$\begin{aligned} \Gamma _{(\alpha , \beta )}:=\{(f(u), f(v))\,|\, \exists a, b \in X \,\,\mathrm{s.t.}\,\, \alpha =f(a), \beta =f(b), \varphi (a, b) = (u,v)\} \end{aligned}$$

If we let $v:= f(u_0), w:= f(v_0)$, then $(v, w) \in \Gamma _{(\alpha , \beta )}$. We define $\psi : Y^2 \rightarrow Y^2$ by $\psi (\alpha , \beta ) := (v, w)$. Then it is well-defined. In fact, if we assume $\psi (\alpha , \beta ) = (v, w)$ and $\psi (\alpha , \beta )= (p, q)$, then there exist $u_1, v_1 \in X$ such that $p= f(u_1), q= f(v_1)$ and $(p, q)\in \Gamma _{(\alpha , \beta )}$. It follows that $\varphi (a, b) = (u_1, v_1)$ and $\varphi (a, b) = (u_0, v_0)$. Since $\varphi$ is a mapping, we obtain $p=v, q= w$, which proves that $\psi : Y^2 \rightarrow Y^2$ is a mapping.

Given $\gamma =f(c)\in Y$, since *f* is an epimorphism, we have

$$\begin{aligned} \alpha \bullet (\beta \bullet \gamma ) & =   f(a)\bullet (f(b) \bullet f(c))\\ & =   f(a*(b*c))\\ &=  f((u_0*v_0)*c)\\ &= (f(u_0)\bullet f(v_0))\bullet f(c)\\ & =   (v\bullet w)\bullet \gamma . \end{aligned}$$

This proves that $(Y, \bullet )$ is a left-twisted semigroup with respect to a map $\psi : Y^2\rightarrow Y^2$. $\square$

Let $(X,*)$ and $(Y,\bullet )$ be groupoids. A map $f: (X,*)\rightarrow (Y,\bullet )$ is said to be *left-twisted-injective* if $f(a*(b*c)) = f((u*v)*c)$, then there exist $u^{\prime }$ and $v^{\prime }$ in *X* such that $a*(b*c) = (u^{\prime }*v^{\prime })*c$ and $f(u)= f(u^{\prime }), f(v)= f(v^{\prime })$ where $a, b, c, u, v\in X$. For example, the canonical group homomorphism $\pi : G\rightarrow G/N$ is left-twisted-injective.

### **Proposition 11**

*Let* $(Y, \bullet )$ *be a left-twisted semigroup with respect to a map* $\psi$. *If* $f: (X,*)\rightarrow (Y, \bullet )$ *is a left-twisted-injective epimorphism, then there exists a map* $\varphi : X^2\rightarrow X^2$ *such that* $(X, *)$ *is a left-twisted semigroup with respect to the map* $\varphi$.

### *Proof*

For any $a, b, c\in X$, since $f:X\rightarrow Y$ is onto, there exist $\alpha , \beta , \gamma \in Y$ such that $f(a) = \alpha , f(b) = \beta , f(c) = \gamma$. Since $(Y,\bullet )$ is a left-twisted semigroup, there exist $v, w\in Y$ such that $\alpha \bullet (\beta \bullet \gamma ) = (v\bullet w)\bullet \gamma$ and $\psi (\alpha , \beta ) = (v, w)$. Since *f* is onto, there exist $p, q\in X$ such that $f(p)= v, f(q) = w$ and hence $f(a)\bullet (f(b)\bullet f(c)) = (f(p)\bullet f(q))\bullet f(c)$. Since *f* is left-twisted-injective, we have

13 $$\begin{aligned} a*(b*c) = (p^{\prime }*q^{\prime })*c \end{aligned}$$

for some $p^{\prime }, q^{\prime }\in X$ where $f(p) = f(p^{\prime }), f(q) = f(q^{\prime })$. Given $a, b\in X$, we obtain $p^{\prime }, q^{\prime }\in X$ satisfying (13), which means that $p^{\prime }, q^{\prime }\in X$ are determined by choosing $a, b\in X$, i,e., there exists a map $\varphi : X^2\rightarrow X^2$ such that $\varphi (a, b) = (p^{\prime }, q^{\prime })$. Hence $(X,*)$ is also a left-twisted semigroup with respect to $\varphi$. $\square$

In Theorem 3, given $a, b, c\in X$, there exist $u, v\in X$ such that $a*(b*c)= (u*v)*c$ where $\varphi (a, b) = (u, v)$. Since $f: (X, *)\rightarrow (Y, \bullet )$ is an epimorphism, we have $f(a)\bullet (f(b)\bullet f(c))= f(a*(b*c)) = f((u*v)*c) = (f(u)\bullet f(v))\bullet f(c)$. Now, since $(Y,\bullet )$ is a left-twisted semigroup with respect to $\psi$, we obtain $p, q\in Y$ such that $f(a)\bullet (f(b)\bullet f(c)) = (p\bullet q)\bullet f(c)$ where $\psi (f(a), f(b))= (p, q)$. It follows that $(p\bullet q)\bullet f(c)=(f(u)\bullet f(v))\bullet f(c)$.

Using the notion of this concept, we introduce an equivalence relation on the Cartesian product $Y\times Y$ of any groupoid $(Y, \bullet )$ (not necessarily a (left-twisted) semigroup). Let $(X,*)$ and $(Y,\bullet )$ be groupoids and let $f:X\rightarrow Y$ be a map. Define a relation “$\equiv$” on $Y^2$ using *f* by

14 $$\begin{aligned} (\alpha , \beta ) \equiv (\gamma , \delta ) (\hbox {mod}\, f) \Longleftrightarrow (\alpha \bullet \beta )\bullet f(x)= (\gamma \bullet \delta )\bullet f(x), \quad  \forall \quad x\in X \end{aligned}$$

where $\alpha , \beta , \gamma , \delta \in Y$. Then it is easy to show that $\equiv (\hbox {mod}\, f)$ is an equivalence relation on $Y^2$. Hence $Y^2$ is partitioned into equivalence classes $[(\alpha , \beta )]:= \{(\gamma , \delta )\,|\, (\alpha , \beta ) \equiv (\gamma , \delta ) (\hbox {mod}\, f)\}$.

### *Example 8*

Consider a *d*-algebra $(X,*, 0)$ and a *BCK*-algebra $(Y,\bullet , 0)$ as follows:

If we define a map $f: X\rightarrow Y$ by $f(0)= 0, f(1)= b, f(2)=a$, then $Y^2 = \{[(0,0)], [(a,0)], [(b, a)]\}$ where $[(0, 0)] =\{(0, 0), (0, a), (0, b), (a, a),$ $(b, b)\}$, $[(a, 0)]=\{(a, 0), (a, b)\}, [(b, a)]=\{(b, a), (b, 0)\}$.

Thus, let $f: (X,*)\rightarrow (Y,\bullet )$ be an epimorphism of groupoids, where $(X,*)$ and $(Y,\bullet )$ are left-twisted semigroups with respect to $\varphi$ and $\psi$ respectively, and let $\varphi (a, b) = (u, v)$ for some $u, v\in X$ and $\psi (f(a), f(b))= (p, q)$ for some $p, q\in Y$. Then $a*(b*c) = (u*v)*c$ and $f(a)\bullet (f(b)\bullet \gamma ) = (p\bullet q)\bullet \gamma$ for any $c\in X, \gamma \in Y$. Since *f* is an epimorphism, we have $f(a)\bullet (f(b)\bullet f(c)) = (f(u)\bullet f(v))\bullet f(c)$ and hence $(f(u)\bullet f(v))\bullet \gamma = (p\bullet q)\bullet \gamma$ for any $\gamma \in Y$. It follows that $(f(u), f(v))\equiv (p, q)(\hbox {mod}\, f)$. We summarize:

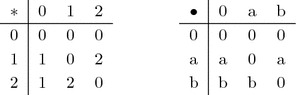



### **Proposition 12**

*Let* $f: (X,*)\rightarrow (Y, \bullet )$ *be an epimorphism of groupoids, where* $(X,*)$ *and* $(Y,\bullet )$ *are left-twisted semigroups with respect to* $\varphi$ *and* $\psi$ *respectively. If* $\varphi (a, b) = (u, v)$, *then* $\psi (f(a), f(b)) \equiv (f(u), f(v)) (\hbox {mod}\, f)$.

Given an epimorphism $f: (X,*)\rightarrow (Y,\bullet )$ as in Proposition 12, we define a new map $\widetilde{f}: X^2\rightarrow Y^2$ by $\widetilde{f}(x_1, x_2):=(f(x_1), f(x_2)), \forall x_1, x_2\in X$. Using this map we obtain $(\psi \circ \widetilde{f})(a, b) = \psi ( \widetilde{f}(a, b))=\psi (f(a), f(b)))= (p, q)$ and $(\widetilde{f}\circ \varphi )(a,b) = \widetilde{f}(\varphi (a, b)))= \widetilde{f}(u, v) = (f(u), f(v))$ where “$\circ$” is a composition of functions. Hence $\psi \circ \widetilde{f}(a, b) \equiv \widetilde{f}\circ \varphi (a, b) (\hbox {mod}\, f)$, i.e., the following diagram is a commuting diagram:

For the other types of twisted semigroups we have similar situations. In the case of a dual left-twisted semigroup, we obtain the following results.

**Theorem 3** $^{\prime }$. *Let* $(X, *)$ *be a dual left-twisted semigroup with respect to a map* $\varphi : X^2\rightarrow X^2$. *If* $f: (X,*)\rightarrow (Y, \bullet )$ *is an* *epimorphism of groupoids, i.e.,* $f(x*y)= f(x)\bullet f(y), \forall x, y\in X$, *then there exists a map* $\psi : Y^2\rightarrow Y^2$ *such that* $(Y, \bullet )$ *is a dual left-twisted semigroup with respect to the map* $\psi$.

Let $(X,*)$ and $(Y,\bullet )$ be groupoids. A map $f: (X,*)\rightarrow (Y,\bullet )$ is said to be *dual left-twisted-injective* if $f((a*b)*c)) = f(u*(v*c))$, then there exist $u^{\prime }$ and $v^{\prime }$ in *X* such that $(a*b)*c = u^{\prime }*(v^{\prime }*c)$ and $f(u)= f(u^{\prime }), f(v)= f(v^{\prime })$ where $a, b, c, u, v\in X$.

**Proposition 11** $^{\prime }$. *Let* $(Y, \bullet )$ *be a dual left-twisted semigroup with respect to* $\psi$. *If* $f: (X,*)\rightarrow (Y, \bullet )$ *is a dual left-twisted-injective epimorphism, then there exists a map* $\varphi : X^2\rightarrow X^2$ *such that* $(X, *)$ *is a dual left-twisted semigroup with respect to the map* $\varphi$.

The corresponding relationship on $Y^2$ is now the following. Let $(X,*)$ and $(Y,\bullet )$ be groupoids and let $f:X\rightarrow Y$ be a map. Define a relation “$\equiv$” on $Y^2$ using *f* by

15 $$\begin{aligned} (\alpha , \beta ) \equiv (\gamma , \delta ) (\hbox {mod}\, f)^{*} \Longleftrightarrow \alpha \bullet (\beta \bullet f(x))= \gamma \bullet (\delta \bullet f(x)), \forall x\in X \end{aligned}$$

where $\alpha , \beta , \gamma , \delta \in Y$. Then it is easy to show that $\equiv (\hbox {mod}\, f)^{*}$ is an equivalence relation on $Y^2$. Hence $Y^2$ is partitioned into equivalence classes $[(\alpha , \beta )]^{*}:= \{(\gamma , \delta )\,|\, (\alpha , \beta ) \equiv (\gamma , \delta ) (\hbox {mod}\, f)^{*}\}$.

The other four types go in entirely the same way. We list the relevant information.

(1): *right-twisted semigroups*: $\psi (\alpha , \beta )$ is defined as previous cases, and the equivalence relation on $Y^2$ is $(\alpha , \beta ) \equiv (\gamma , \delta ) [\hbox {mod}\, f]$ provided for all $x\in X$, $f(x)\bullet (\alpha \bullet \beta ) = f(x)\bullet (\gamma \bullet \delta )$.
(2): *dual right-twisted semigroups*: $\psi (\alpha , \beta )$ is defined as previous cases, and the equivalence relation on $Y^2$ is $(\alpha , \beta ) \equiv (\gamma , \delta ) [\hbox {mod}\, f]^{*}$ provided for all $x\in X$, $(f(x)\bullet \alpha )\bullet \beta = (f(x)\bullet \gamma )\bullet \delta$.
(3): *middle-twisted semigroups*: $\psi (\alpha , \beta )$ is defined as previous cases, and the equivalence relation on $Y^2$ is $(\alpha , \beta ) \equiv (\gamma , \delta ) <\hbox {mod}\, f>$ provided for all $x\in X$, $\alpha \bullet (f(x)\bullet \beta ) = \gamma \bullet (f(x)\bullet \delta )$.
(4): *dual middle-twisted semigroups*: $\psi (\alpha , \beta )$ is defined as previous cases, and the equivalence relation on $Y^2$ is $(\alpha , \beta ) \equiv (\gamma , \delta ) <\hbox {mod}\, f>^{*}$ provided for all $x\in X$, $(\alpha \bullet f(x))\bullet \beta = (\gamma \bullet f(x))\bullet \delta$.

Given one of the six types of twisted semigroups, let $\{(X_{\alpha }, *_{\alpha }, \varphi _{\alpha })\}_{\alpha \in I}$ be an indexed family of one of these types of twisted semigroups. Let $X=\prod _{\alpha \in I}X_{\alpha }$ be the direct product and let $\pi _{\alpha }: X \rightarrow X_{\alpha }$ be the canonical surjection. Let *X* be equipped with the product binary operation given by the formula $(x_{\alpha })*(y_{\alpha }) = (x_{\alpha }*_{\alpha } y_{\alpha })$. If we define a map $\varphi ((a_{\alpha }), (b_{\alpha })) = (\varphi _{\alpha }(a_{\alpha }, b_{\alpha }))= ((u_{\alpha }, v_{\alpha }))$, then it follows that $(X,*)$ is a twisted semigroup of the same type with respect to the map $\varphi$.

In order to obtain varieties we must be able to claim that if $(X,*)$ is a twisted semigroup of a given (one of the six) type(s) with respect to a map $\varphi$ and if $(A,*)$ is a subgroupoid of $(X,*)$, then it is also of the same type, i.e., there is a function $\psi : A^2 \rightarrow A^2$ (rather than $\psi : A^2 \rightarrow X^2, \psi =$ ${\varphi \mid A^2}$) which satisfies the required identity belonging to the special type in question. We give a counter-example that *a subgroupoid* $(A,*)$ *of a dual left-twisted semigroup* $(X,*)$ *need not be a dual left-twisted semigroup*.

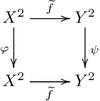



### *Example 9*

In Example 4, let *Z* be the collection of all integers. Then $(Z,*)$ is a subgroupoid of $(\mathbf{R},*)$ and $(\mathbf{R}, *)$ is a dual left-twisted semigroup with respect to $\varphi$. If we let $a:=13, b:=12$, then $v^3= 13(13-12)(v^2-1)=13(v^2-1)$. Assume *v* is an integer. Then $v= 13k$ for some integer *k*. This means that $(13)^2k^2(1-k) = 1$, which is impossible for an integer *k*. Hence there is no mapping $\varphi : Z^2 \rightarrow Z^2$ such that $(Z,*)$ is a dual left-twisted semigroup with respect to $\varphi$.

## Conclusions

In this paper, we introduced the concept of several types of groupoids related to semigroups, viz., twisted semigroups for which twisted versions of the associative law hold. Besides a number of examples and a discussion of homomorphisms, a class of groupoids of interest was the class of groupoids defined over a field $(X,+, \cdot )$ via a formula $x*y=\lambda x + \mu y$, with $\lambda , \mu \in X$, fixed structure constants. Properties of these groupoids as twisted semigroups were discussed with several results of interest obtained, e.g., that in this setting simultaneous left-twistedness and right-twistedness of $(X,*)$ implies the fact that $(X,*)$ is a semigroup.

In the investigation of “residual associativity” in groupoids one encounters a number of levels. The strongest version of such residual associativity is “associativity” itself. Making a study of twisted semigroups, besides being of interest in itself, is also relevant in several other ways. One may wish to determine as precisely as possible how different twisted semigroups may differ from semigroups. Accordingly we note that the study of twisted semigroups, commenced in this paper, will prove to be a rich area for research both in itself and as embedded in the area of the study of “general theory of groupoids”.
